# Whole-Genome Sequencing of Six Strains of Salmonella enterica Isolated from Imported Meat in Algeria

**DOI:** 10.1128/MRA.00615-19

**Published:** 2019-08-29

**Authors:** A. Deriet, M. Berrazeg, S. C. J. De Keersmaecker, N. Botteldoorn, K. Vanneste, B. Verhaegen, N. H. C. Roosens, F. Mouffok, R. Drali

**Affiliations:** a Unité environnement, Laboratoire de Bactériologie des Aliments, Eaux et Environnement, Institut Pasteur d'Algérie, Alger, Algeria; b Laboratoire de Microbiologie, Institut Pasteur d'Algérie, Antenne d'Oran, Oran, Algeria; c Département de Biologie, Faculté des Sciences de la Nature et de la Vie, Université d'Oran 1, Oran, Algeria; d Sciensano, Transversal Activities in Applied Genomics, Brussels, Belgium; e Sciensano, Service Food Borne Pathogens, Brussels, Belgium; Broad Institute

## Abstract

Nontyphoidal *Salmonella* (NTS) is one of the main causes of foodborne disease worldwide. In this report, we announce the first whole-genome sequencing of six strains of Salmonella enterica isolated from imported meat in Algeria. The genome sizes ranged from 4,601,209 to 4,958,962 bp. Antimicrobial resistance (AMR) genes, plasmids, and virulence factors were detected.

## ANNOUNCEMENT

Nontyphoidal *Salmonella* (NTS) remains one of the most important foodborne pathogens worldwide and presents a big challenge to public health and food safety (1). Meat constitutes a major source of human *Salmonella* infections (2). Globalization and the import of food, especially meat, have increased the risk linked to NTS (3, 4).

Few studies describing NTS of clinical or environmental origin have been reported in Algeria, just as, to the best of our knowledge, no *Salmonella* genome from Algeria has been sequenced (5). In the context of the microbiological surveillance of imported food products, 33 NTS strains were isolated during the period from 2013 to 2014 according to ISO 6579 (6). Thus, buffered peptone water was used for the first enrichment, and then selenite cysteine broth and Rappaport-Vassiliadis broth were used for selective enrichment. Finally, xylose lysine deoxycholate (XLD) and Hecktoen agar were used to help isolate *Salmonella* strains. Biochemical identification and serotyping of *Salmonella* strains were performed using the API 20E test system (bioMérieux) and the White-Kauffmann-Le Minor scheme, respectively (7). Six strains isolated from frozen meats, belonging to five different serotypes from three countries (Brazil, Denmark, and India), were selected for genomic investigations (Table 1). Genomic DNA was extracted using the Genomic-tip 20/G kit (Qiagen) following the manufacturer’s instructions. A paired-end 2 × 250-bp sequencing run was performed using an Illumina MiSeq system. The Nextera XT DNA library preparation kit was used to construct libraries from the extracted DNA. Raw sequence reads were trimmed using Trimmomatic v0.36.4 with the following options: trailing, 10; leading, 10; slidingwindow, 4:20; and minlen, 40 (8). Assembly was carried out using Spades v1.3.1 with default settings (9). Annotation of assembly was done using Prokka rapid prokaryotic genome annotation v1.11 with default settings (10). Antimicrobial resistance (AMR) gene occurrence was investigated with ResFinder v3.1 with default settings (https://cge.cbs.dtu.dk/services/ResFinder/). The presence of plasmid replicons was explored using PlasmidFinder v2.0 with default settings (https://cge.cbs.dtu.dk/services/PlasmidFinder/). Virulence was investigated using the virulence factors database (http://www.mgc.ac.cn/VFs).

**TABLE 1:** Metadata of the 6 strains of Salmonella enterica isolated from frozen meat

Strain	Origin	Yr	ST^*a*^	Serotype	Plasmid (size [bp])	Genome size (bp)	No. of reads	No. of contigs	Genome coverage (×)	No. of CDS^*b*^	No. of contigs of >1,000 bp	GC content (%)	*N*_50_ (bp)	No. of tRNAs	Assembly accession no.	SRA accession no.
1	Denmark	2014	185	Senftenberg		4,927,366	807,760	145	26	4,622	48	52.01	261,753	74	GCA_006349135	SRR9099608
2	India	2014	972	Berlin		4,601,209	559,190	132	18	4,267	47	52.18	176,650	64	GCA_006349045	SRR9099598
3	India	2013	365	Weltevreden	IncFII(S) (81,966)	4,958,962	1,101,862	212	39	4,598	85	52.14	143,538	68	GCA_006349035	SRR9099599
4	India	2014	14	Senftenberg		4,862,749	1,440,094	168	51	4,519	55	52.01	262,096	80	GCA_006349055	SRR9099600
5	India	2014	11	Enteritidis	IncFIB(S) (59,372)	4,700,259	893,472	94	32	4,422	33	52.12	311,934	73	GCA_006348995	SRR9099601
6	Brazil	2013	82	Munchen		4,823,913	1,339,050	213	47	4,501	43	52.08	258,398	75	GCA_006349105	SRR9099602

a ST, sequence type.

b CDS, coding sequences.

An average of 1,023,571 reads of 250 bp per strain was obtained. The genome sizes ranged from 4,601,209 to 4,958,962 bp with an average coverage depth of 35.5×. The AMR gene *aac(6′)-Iaa* was found in all the strains, while *qnrB19* was found only in strain 6. Sequenced *Salmonella* strains contained an average of 161 ± 9 virulence factors, while all contained the *Salmonella* pathogenicity islands SPI-1 and SPI-2.

Two plasmids with 100% identity with published plasmids were identified using BLASTN on the NCBI database. These are the plasmids characterized in Salmonella enterica serovar Weltevreden strain 1655 (GenBank accession number CP014997) and Salmonella enterica serovar Enteritidis strain OLF-SE3-98983-4 (CP011843), found in strains 3 and 5 respectively (Table 1).

This study highlighted the infection risk related to imported meat and provided the first genome sequences of *Salmonella* spp. in Algeria, which should allow comparison and monitoring of *Salmonella* infections.

### Data availability.

Genomic sequences have been deposited in the NCBI Sequence Read Archive (PRJNA544053) and NCBI GenBank (BioProject number PRJNA540702).
